# Activation of Multidimensional Defenses in *Camptotheca acuminata* Seedlings Against *Spodoptera frugiperda* Larvae

**DOI:** 10.3390/plants15121796

**Published:** 2026-06-11

**Authors:** Wenhui Ma, Chunhao Chang, Jianing Cheng, Yanyan Wang, Xiaoxiao Gao, Fang Yu

**Affiliations:** 1 School of Biological Engineering, Dalian Polytechnic University, Dalian 116034, China; mwh24630@163.com (W.M.); wang_yy@dlpu.edu.cn (Y.W.); 2 College of Horticulture and Forestry, Tarim University, Alar 843300, China; doc_cch@taru.edu.cn; 3 College of Bioscience and Biotechnology, Shenyang Agricultural University, Shenyang 110866, China; chengjianing@syau.edu.cn

**Keywords:** *Camptotheca acuminata*, *Spodoptera frugiperda*, biotic stress, camptothecin, plant–herbivore interactions

## Abstract

*Camptotheca acuminata*, the primary botanical source of camptothecin (CPT), employs this monoterpenoid indole alkaloid as a key chemical defense against herbivores in addition to its established clinical pharmaceutical importance. Given that *Spodoptera frugiperda* infestations pose a severe threat to *C. acuminata* seedlings, we examined integrated, multi-layered defense mechanisms that combine physical barriers with chemical toxins to bolster plant resistance. Physiological analyses revealed that herbivory induces antioxidant enzymes such as superoxide dismutase (SOD) and catalase (CAT), alongside broader metabolic reprogramming. These responses are orchestrated by differential activation of jasmonic acid (JA) and salicylic acid (SA) signaling pathways, which together drive complex defense mobilization, including a marked increase in trichome density. Concurrently, insect herbivory activates the MYB-bHLH-WD40 (MBW) transcriptional complex to promote trichome development while upregulating core CPT biosynthetic genes. In particular, two cytochrome P450 genes, *Ca32236* and *CaCYP81BQ18*, mediate the accumulation of 10-hydroxycamptothecin (10-HCPT), a derivative that is sparingly soluble in water, which enables alkaloid transport and sequestration to specialized storage sites, including trichomes. Collectively, these stress-responsive strategies confer potent insecticidal activity against *S. frugiperda* and provide valuable insights for improving protection in *C. acuminata* seedling plantations.

## 1. Introduction

Since the transition of their ancestors from aquatic to terrestrial habitats, plants have continuously co-evolved with a diverse array of herbivorous threats. To cope with their sessile lifestyle and the absence of an adaptive immune system, plants have evolved sophisticated, multi-layered defense mechanisms capable of detecting and counteracting these biotic stresses [1,2]. Herbivory is perceived through both mechanical stimuli, such as biting and piercing, and chemical cues released by herbivore-derived compounds, including oral secretions, regurgitants, frass, and oviposition fluids [3]. These perception events initiate complex signaling cascades that coordinate direct and indirect defense strategies. Direct defenses comprise physical barriers (e.g., spines, trichomes, and cuticular wax) as well as chemical toxins derived from non-volatile specialized metabolites, whereas indirect defenses are primarily mediated by volatile organic compounds that recruit natural enemies of herbivores [4,5,6]. Fundamentally, the execution of these defense strategies is underpinned by a range of physiological and molecular adjustments, including stomatal regulation, reactive oxygen species (ROS) bursts, antioxidant enzyme activation, lipid peroxidation, and large-scale transcriptional reprogramming [7,8].

*Spodoptera frugiperda* is a highly polyphagous pest that feeds on more than 350 plant species spanning 76 families, inflicting severe damage on over 80 economically important crops, including maize, rice, and soybean, and causing substantial economic losses [9,10]. These impacts are further exacerbated by the insect’s remarkable metabolic adaptability, broad resistance to multiple insecticides, and tolerance to Bt toxins [11,12,13,14], all of which severely complicate control strategies. Upon feeding, larvae release oral secretions containing fatty acid-amino acid conjugates (FACs). Plant perception of these FACs rapidly activates jasmonic acid (JA)-associated pathways and induces the accumulation of defense-related secondary metabolites, including benzoxazinoids, terpenoids, and proteinase inhibitors [4,15,16]. These induced metabolites constitute the primary defense response triggered by FACs. However, insect-derived salivary enzymes (e.g., SfruACY) degrade FACs and attenuate this defense induction, thereby enhancing larval performance on host plants [17].

Plant secondary metabolites constitute core elements of chemical defense systems, equipping plants with robust capabilities that counter biotic stresses. Among these metabolites, camptothecin (CPT), a monoterpenoid indole alkaloid (MIA), serves as a primary defensive compound within evolutionarily conserved pathways across diverse plant taxa, including *Camptotheca* (*Nyssaceae*), *Nothapodytes* (*Icacinaceae*), and *Ophiorrhiza* (*Rubiaceae*) [18,19,20]. CPT and its structural analogs occur in 43 species spanning eight orders [20], including the medicinal tree *Camptotheca acuminata* and the herb *Ophiorrhiza pumila*. Notably, *C. acuminata* is a deciduous tree species endemic to southern China and is widely cultivated as an ornamental and medicinal plant [21]. Beyond serving as the primary natural source of camptothecin (CPT), which is the precursor of the anticancer drugs irinotecan and topotecan, *C. acuminata* also plays important economic and ecological roles in industrial oil production, papermaking, furniture manufacturing, and environmental remediation [22]. *S frugiperda*, a highly polyphagous pest native to the Americas, invaded China in 2018. The absence of natural enemies and favorable climatic conditions have facilitated its rapid spread across major agricultural regions [23]. Although maize is its primary host, the pest’s broad host range poses a potential threat to young *C. acuminata* seedlings even with their CPT-related chemical defenses. This risk warrants further monitoring.

Despite the well-established role of CPT in plant defense, the underlying metabolic basis of CPT-mediated protective responses remains poorly understood. In *C. acuminata*, CPT biosynthesis commences with the condensation of tryptamine and secologanin to yield the key precursor strictosidinic acid [24]. However, the downstream steps in the CPT biosynthetic pathway remain incompletely characterized [20,25]. Following its synthesis, CPT is hydroxylated to 10-hydroxycamptothecin (10-HCPT) by cytochrome P450 enzymes (CYP450s) [26]. Due to its increased water solubility, 10-HCPT contributes to the systemic distribution of chemical defenses, and may fulfill distinct defensive functions. Nevertheless, the regulatory mechanisms controlling the conversion of CPT to 10-HCPT by CYP450s remain unclear. This knowledge gap is further compounded by the high degree of tissue specificity in alkaloid accumulation.

In *C. acuminata*, CPT concentrations in young leaves are five to six times higher than in mature tissues [27], while seedlings display peak production levels relative to later developmental stages. This spatiotemporal distribution pattern indicates an adaptive strategy for safeguarding vulnerable tissues, such as young leaves and seedlings, against herbivory. At the cellular level, glandular trichomes and specialized idioblasts serve as the primary storage sites for CPT [28,29,30], with trichome density exhibiting a positive correlation with CPT concentration [29]. Such associations suggest that physical barriers and chemical defenses may be co-regulated through a common transcriptional network in response to herbivore stress. Consequently, deciphering the regulatory networks that control CPT biosynthesis and their integration with physical barriers like glandular trichomes is crucial for understanding how plants optimize defensive resource allocation under herbivore pressure.

To address these knowledge gaps, the present study investigates the responses of *C. acuminata* seedlings to *S. frugiperda* larvae, with a focus on elucidating the regulatory networks governing CPT biosynthesis, its hydroxylation to 10-HCPT, and its coordination with physical defenses such as trichomes. By integrating physiological response, transcriptional analysis, and metabolite profiling, we aim to elucidate signaling pathways (e.g., JA and SA) that modulate plant defense to herbivore stresses in *C. acuminata*. These insights will not only enhance our understanding of plant–herbivore interactions in *C. acuminata* but also provide a foundation for developing sustainable pest management strategies and optimizing CPT production for pharmaceutical applications.

## 2. Results

### 2.1. Physiological and Defensive Responses to S. frugiperda Herbivory

**Figure 1:**
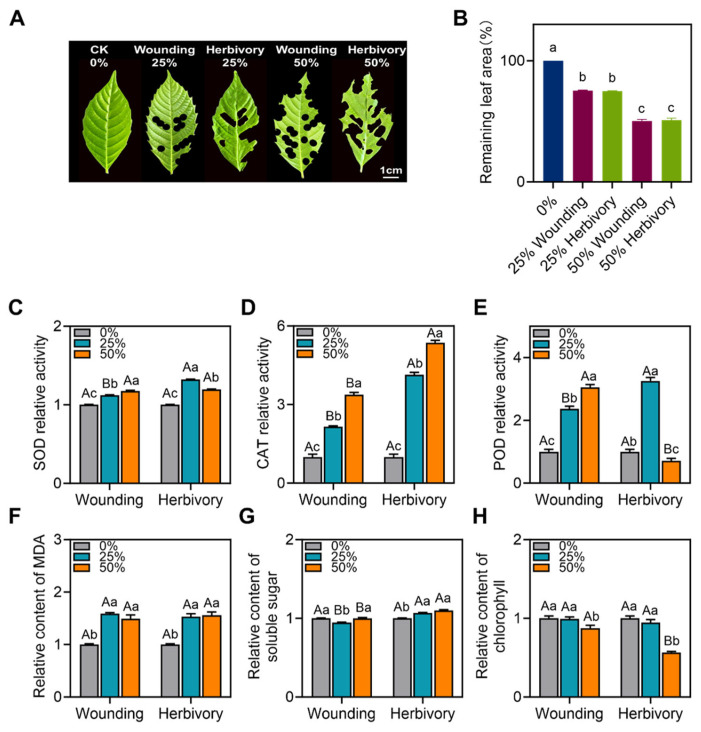
*S. frugiperda* herbivory-induced physiological changes in *C. acuminata* seedling leaves. (**A**) Representative leaves under untreated control plants caged without intervention (CK), mechanical wounding, and herbivory treatments (25% and 50% leaf area damage). Bar = 1 cm. (**B**) Damaged area was calculated using ImageJ 1.44p. (**C**) Superoxide dismutase (SOD) activity. (**D**) Catalase (CAT) activity. (**E**) Peroxidase (POD) activity. (**F**) Malondialdehyde (MDA) content. (**G**) Soluble sugar content. (**H**) Chlorophyll content. Data were analyzed using two-way ANOVA followed by Tukey’s multiple comparisons test (*p* < 0.05). Different lowercase letters indicate significant differences among damage levels within the same treatment group, whereas different uppercase letters indicate significant differences between treatment types at the same damage level. Data are presented as mean ± SD (*n* = 3 independent biological replicates, each consisting of pooled samples from 10 plants).

To evaluate the physiological responses of *C. acuminata* to insect herbivory, we subjected plants to two damage levels (Figure 1A). Leaf area loss reached approximately 25% after about 12 h of feeding and 50% after about 24 h, the points at which samples were collected (Figure 1A,B). In parallel, mechanical wounding with a hole puncher was conducted to distinguish insect-specific signaling from mere physical injury. Antioxidant enzyme activities, including superoxide dismutase (SOD), Catalase (CAT), and peroxidase (POD), were subsequently measured using NBT reduction, H_2_O_2_ decomposition, and guaiacol oxidation assays, respectively, as markers of systemic defense activation. Under insect herbivory, SOD activity increased significantly, reaching 1.3-fold and 1.2-fold of control (untreated control plants caged without intervention) levels at the 25% and 50% damage stages, respectively (Figure 1C). CAT activity exhibited a more substantial elevation, rising to 4.0-fold and 5.2-fold of control under the corresponding damage levels (Figure 1D). Conversely, POD activity displayed distinct patterns. At 25% leaf area loss, POD activity differed significantly among these treatments, whereas at 50% leaf area loss, mechanically wounded plants exhibited significantly higher POD activity than herbivore-treated and control plants (Figure 1E). These observations suggest that insect-specific elicitors, likely derived from oral secretions, regulate POD activity, with pronounced suppression emerging under heightened feeding intensity.

Malondialdehyde (MDA), a key marker of lipid peroxidation and oxidative membrane injury during biotic stress, accumulated progressively with increasing leaf damage in both treatments, with no significant differences detected between insect herbivory and mechanical wounding (Figure 1F). This outcome suggests that the degree of oxidative lipid peroxidation depends mainly on the severity of physical tissue disruption rather than on amplification by insect-specific elicitors. Furthermore, soluble sugar content exhibited distinct treatment-dependent patterns (Figure 1G). Soluble sugar accumulation was significantly higher under herbivory than under mechanical wounding at both the 25% and 50% damage levels. Relative to the control, herbivory consistently induced a significant increase in soluble sugar across both damage levels. Conversely, mechanical wounding failed to induce such an accumulation, with no significant increase observed at the 50% damage level. Meanwhile, chlorophyll content remained stable at 25% damage but decreased significantly at 50% damage under both treatments (Figure 1H). Notably, at the 50% damage level, the reduction in chlorophyll was significantly more severe under herbivory than under mechanical wounding. Together, these results reveal that herbivory by *S. frugiperda* induces a range of physiological responses in *C. acuminata* seedlings, including shifts in antioxidant capacity and targeted metabolic reprogramming.

### 2.2. Activation of Hormonal Signaling and Trichome-Mediated Physical Defense

**Figure 2:**
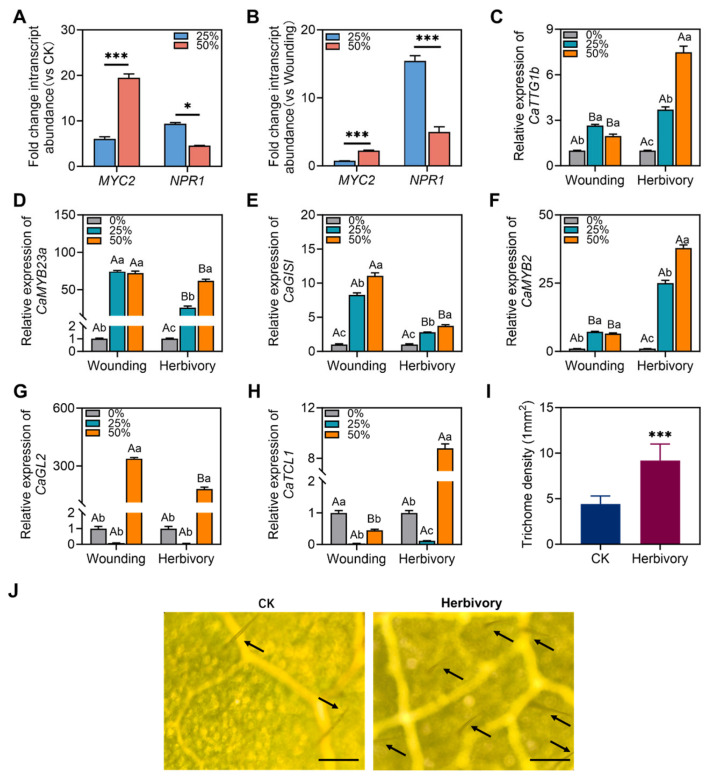
*S. frugiperda* herbivory activates hormone signaling and promotes trichome development in *C. acuminata*. (**A**,**B**) Relative expression of the JA pathway key regulator *MYC2* and the SA pathway response gene *NPR1*. (**C**–**H**) Relative expression levels of trichome-associated regulatory genes. (**I**) Trichome density on the leaves of the CK and herbivory plants. (**J**) Representative microscopic images of newly formed trichomes on leaves under control (CK) and herbivory treatments. Black arrows indicate trichomes. Bar = 0.1 mm. Data for (**C**–**H**) were analyzed using two-way ANOVA followed by Tukey’s multiple comparisons test (*p* < 0.05). Different lowercase letters indicate significant differences among damage levels within the same treatment group, whereas different uppercase letters indicate significant differences between treatment types at the same damage level. For (**A**,**B**), pairwise comparisons between the 25% and 50% damage levels within individual genes were evaluated using Student’s *t*-test. The comparison between CK and herbivory in (**I**) was also evaluated using Student’s *t*-test. Statistical significance is indicated by asterisks (* *p* < 0.05, *** *p* < 0.001). Data are presented as mean ± SD (*n* = 3 independent biological replicates, each consisting of pooled samples from 10 plants).

To assess the early activation of defense signaling, we analyzed the expression of JA and SA marker genes. Real-time qPCR revealed that *CaMYC2*, a core JA signaling component, increased progressively with damage severity, reaching 6.0-fold at 25% damage and 19.5-fold at 50% damage. In contrast, the expression of the SA marker *CaNPR1* was highest with 25% damage (9.4-fold increase), whereas it was lower (4.6-fold) in the 50% damage group (Figure 2A). Comparable expression patterns were observed in comparisons relative to the mechanical wounding treatment (Figure 2B). These results indicate that both JA and SA signaling pathways are engaged in the response to herbivory but exhibit distinct temporal dynamics as feeding intensity increases.

Given that JA signaling is known to regulate trichome initiation [31,32], we next examined whether the observed hormonal activation coincided with the induction of trichome-mediated physical defenses. The expression of *CaTTG1b* and *CaMYB23a*, which encode core components of the MYB-bHLH-WD40 (MBW) transcriptional complex that regulates trichome development and defense responses, was significantly upregulated under insect herbivory (Figure 2C,D). Notably, *CaMYB23a* (encoding an R2R3-MYB transcription factor that determines target gene specificity within the MBW complex) showed a 61.6-fold increase at the 50% damage stage. Additional positive regulators, including *CaGIS1* (encoding a putative HD-ZIP transcription factor involved in trichome initiation) (Figure 2E) and *CaMYB2* (encoding a positive regulator of trichome elongation) (Figure 2F), were also coordinately induced, leading to substantial upregulation of the master regulator of trichome development, *CaGL2* (Figure 2G), which directs trichome cell fate and differentiation. Conversely, the repressor *CaTCL1* showed reduced expression at the 25% damage level but was upregulated 8.1-fold at the 50% damage level (Figure 2H). Microscopic observations confirmed that these molecular alterations were accompanied by a substantial increase in trichome density in newly developed leaves two weeks post-treatment (Figure 2I,J), thereby implying a potential association between JA-mediated signaling and the bolstering of both physical and chemical defenses against herbivory.

### 2.3. Induction of CPT and 10 HCPT Biosynthesis and the Synthesis Transport Storage Model

**Figure 3:**
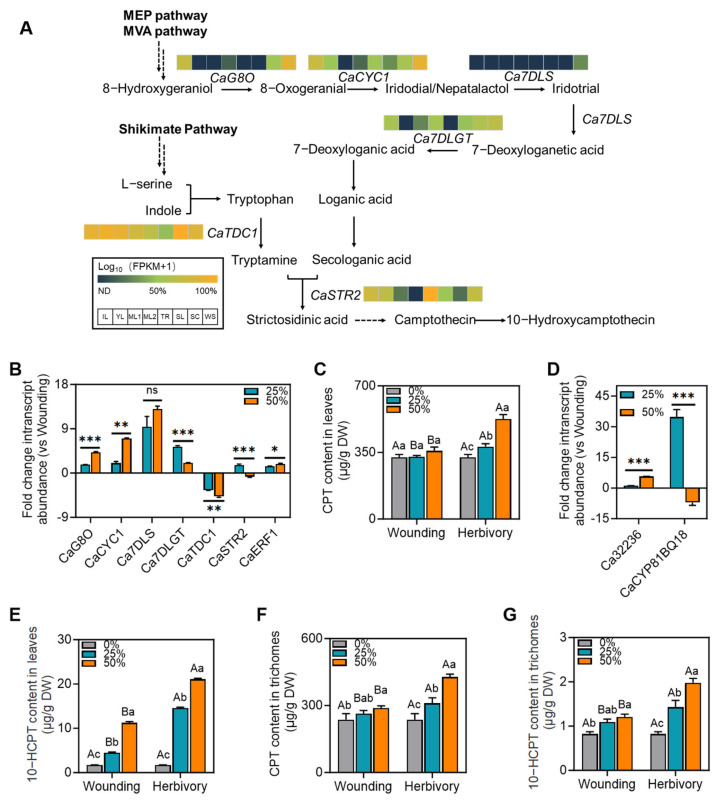
*S. frugiperda* enhances CPT and 10-HCPT biosynthesis in *C. acuminata*. (**A**) Complete map of CPT biosynthetic pathway and expression of pathway enzyme genes in different tissues of *C. acuminata*. Tissue abbreviations: IL, immature leaf; YL, young leaf; TR, trichome; SL, seedling leaf; SC, seedling cotyledons; WS, whole seedling; ML1, mature leaf (rep1); ML2, mature leaf (rep2). GenBank accession numbers: *CaG8O*, GACF01095885; *CaCYC1*, KU842378; *Ca7DLS*, GACF01109828; *Ca7DLGT*, GACF01100860; *CaTDC1*, AAB39708; *CaSTR2*, JF508375. Solid arrows indicate confirmed steps, single dashed arrows indicate uncharacterized steps, and double dashed arrows indicate omitted intermediate steps. (**B**) Relative expression levels of key CPT biosynthetic enzyme genes and the transcription factor *CaERF1*. (**C**) CPT accumulation in leaves. (**D**) Relative expression levels of CYP450s. (**E**) 10-HCPT accumulation in leaves. (**F**) CPT accumulation in trichomes. (**G**) 10-HCPT accumulation in trichomes. Data were analyzed using two-way ANOVA followed by Tukey’s multiple comparisons test (*p* < 0.05). Different lowercase letters indicate significant differences among damage levels within the same treatment group, whereas different uppercase letters indicate significant differences between treatment types at the same damage level. For (**B**,**D**), pairwise comparisons between the 25% and 50% damage levels within individual genes were evaluated using Student’s *t*-test, and statistical significance is indicated by asterisks (* *p* < 0.05, ** *p* < 0.01, *** *p* < 0.001, ns, not significant). Data are presented as mean ± SD (*n* = 3 independent biological replicates, each consisting of pooled samples from 10 plants).

To investigate whether herbivory influences camptothecin biosynthesis at the transcriptional level, we examined the expression patterns of key genes involved in the CPT biosynthetic pathway (Figure 3A,B). Relative to mechanical wounding, insect herbivory triggered significant upregulation of several key genes in the CPT biosynthetic pathway (Figure 3B). At the 25% leaf area loss stage, *CaCYC1*, *Ca7DLS*, and *Ca7DLGT*, as well as the transcription factor *CaERF1* [33], were displayed fold inductions of 2.0, 9.4, 5.4, and 1.4, respectively. At the 50% damage stage, *CaCYC1* and *CaG8O* showed further significant increases to 7.0- and 4.2-fold, respectively, whereas *Ca7DLS* remained highly induced without a significant difference between the two damage levels (Figure 3B). Consistent with the observed transcriptional changes, herbivory induced a marked increase in total leaf CPT content, from 324.7 µg/g to 526.1 µg/g, while mechanical wounding elicited no significant change in accumulation (Figure 3C). A larval survival bioassay showed that *S. frugiperda* larvae continuously confined on *C. acuminata* leaves did not survive beyond 24 h, demonstrating that the plant’s strong defense capacity against this herbivory. Given the limited aqueous solubility of CPT and its proposed translocation to trichomes for storage [30,34], we investigated whether herbivory enhances the biosynthesis of 10-HCPT, a more soluble derivative. Two cytochrome P450 genes, *Ca32236* and *CaCYP81BQ18*, which mediate CPT hydroxylation, were significantly upregulated in response to insect herbivory compared with mechanical wounding, exhibiting distinct temporal expression patterns. *CaCYP81BQ18* expression peaked at the 25% damage stage with a 34.8-fold increase, whereas *Ca32236* reached a 5.6-fold increase at the 50% damage stage (Figure 3D), suggesting an herbivory-induced enhancement in the transcriptional capacity for CPT-to-10-HCPT conversion during feeding. Concurrently, compared with mechanical wounding, 10-HCPT accumulation in leaves increased by 3.3-fold and 1.9-fold at the 25% and 50% damage stages, respectively (Figure 3E), with these relative increases exceeding those observed for CPT (Figure 3B). HPLC analysis further revealed that CPT content in isolated trichomes rose from 236.8 µg/g to 427.8 µg/g under herbivory (Figure 3F), and 10-HCPT was significantly enriched in trichomes compared with whole leaf (Figure 3G). Pearson correlation analysis demonstrated a linear relationship between trichome density and the concentrations of both CPT and 10-HCPT (Figure S1).

Gene expression analysis based on the publicly available transcriptomic dataset (NCBI BioProject PRJNA174448) indicated that genes involved in CPT biosynthesis were predominantly expressed in young leaves and remained barely detectable in trichomes (Figure 3A) [24,34,35], implying that trichomes lack the enzymatic machinery for autonomous CPT biosynthesis despite their high alkaloid content. These findings collectively support a defense model in which CPT and 10-HCPT are synthesized in leaf tissues and subsequently accumulated in trichomes, representing an integration of chemical and physical defenses to form an effective barrier against herbivory.

## 3. Discussion

Consistent with the pivotal role of oral secretions in triggering plant immunity, our investigation demonstrates that *S. frugiperda* herbivory elicits a distinct suite of physiological adjustments in *C. acuminata*. These responses differ markedly from those induced solely by mechanical wounding. Herbivory activated the antioxidant enzymes SOD and CAT more robustly than mechanical damage did, suggesting that insect-specific elicitors amplify the antioxidant response, thus counteracting herbivory-induced oxidative stress. Conversely, POD activity remained elevated following mechanical damage but was suppressed under severe herbivory. This divergent response may reflect the action of salivary effectors that specifically target POD, a phenomenon reported in other plant–insect systems [36]. MDA content increased proportionally to leaf area loss irrespective of treatment, indicating that lipid peroxidation in this system primarily stems from physical tissue disruption rather than insect-specific elicitation. Furthermore, soluble sugars significantly increased only under herbivory at the 50 percent damage level, implying active induction by salivary effectors at higher feeding intensities.

These physiological changes are underpinned by the activation of hormonal signaling pathways. Plants differentiate mechanical wounding from insect herbivory through the recognition of herbivore-associated molecular patterns present in oral secretions [37,38]. Damage-associated molecular patterns, such as extracellular ATP and oligogalacturonides, also contribute to JA and SA signaling via specific receptors [28,39,40,41]. In *C. acuminata*, herbivory, but not mechanical wounding alone, induced both the JA and SA pathways, each exhibiting distinct alterations across damage stages (based on independent plant samples). Expression of the JA-responsive regulator *CaMYC2* was positively associated with increasing leaf damage levels, whereas the SA pathway regulator *CaNPR1* showed the highest expression at the 25% leaf damage level and lower expression at the 50% damage level, although it remained elevated relative to the control. This differential response pattern between JA- and SA-associated regulators is consistent with the canonical JA–SA antagonism model [42,43] under continuous herbivory. The dependence of hormonal activation on oral secretions indicates that components within insect saliva, rather than physical injury, drive the observed signaling changes.

Trichomes function as both mechanical and chemical barriers, constituting a primary line of plant defense against herbivores. They store defensive compounds that are liberated upon disruption during herbivore feeding or movement on leaf surfaces [44]. In tomato plants, these structures perceive mechanical stimuli through calcium wave mediated communication, which subsequently activates JA signaling and terpene biosynthesis [45]. This process underscores the fundamental importance of trichome development in establishing both physical and chemical defenses, thereby enabling plants to effectively resist biotic stresses. The MBW transcriptional complex is a well-established central regulator of trichome initiation and development. Within this framework, GLABRA2 acts as a crucial downstream determinant of trichome cell fate [46,47], emphasizing the significance of this regulatory network in mediating plant responses to biotic stresses. In *C. acuminata*, herbivory strongly upregulated the expression of the MBW complex components *CaTTG1b* and *CaMYB23a*, as well as the trichome master regulator *CaGL2*. This upregulation indicates that JA signaling activates trichome development through evolutionarily conserved transcriptional modules. The negative regulator *CaTCL1*, a homolog of the *Arabidopsis* trichome repressor *TRY* [48], exhibited a biphasic expression pattern. It was suppressed at 25 percent leaf damage and then induced 8.1-fold at 50 percent damage level. This pattern suggests a feedback mechanism that fine tunes physical defense allocation in response to escalating damage levels.

Concurrently, herbivory upregulated CPT biosynthetic genes, leading to enhanced accumulation of CPT and its hydroxylated derivative 10-HCPT. JA signaling is known to regulate the biosynthesis of diverse secondary metabolites, including terpenoids, alkaloids, and glucosinolates, by activating downstream transcription factors of the ERF and MYC families [49,50]. Notably, trichome development and alkaloid biosynthesis were closely associated in *C. acuminata*. Trichome density correlated positively with CPT and 10-HCPT contents, and young leaves exhibited higher trichome density and alkaloid levels than mature tissues [29]. This spatial coincidence suggests that physical and chemical defenses are coregulated and colocalized to maximize defensive efficacy. Importantly, these findings indicate that mechanical wounding alone cannot fully reproduce the multidimensional defense responses triggered by insect herbivory, thereby highlighting the critical role of herbivore-specific signals, such as oral secretions, in specifically activating the *C. acuminata* defense system.

A larval survival bioassay showed that *S. frugiperda* larvae continuously confined on *C. acuminata* leaves did not survive beyond 24 h, demonstrating that the plant’s strong defense capacity against this herbivory. Gene expression analysis based on publicly available transcriptomic data analysis revealed that 280 genes involved in CPT biosynthesis, particularly those within the iridoid pathway, are expressed predominantly in leaf internal tissues rather than in trichomes. This expression pattern is consistent with findings in other alkaloid accumulating plants, such as *Nicotiana benthamiana* and *Catharanthus roseus*, where secondary metabolites are synthesized in leaf internal tissues and subsequently transported to surface structures for storage [51,52]. Such spatial separation of synthesis and storage is thought to serve two adaptive functions by preventing autotoxicity in metabolically active leaf cells while simultaneously positioning chemical defenses at the plant–insect interface, where trichomes function as both physical barriers and concentrated toxin reservoirs.

The hydroxylation of CPT to 10-HCPT represents a critical metabolic node that determines the balance between two defensive alkaloids with distinct physicochemical properties. Our study reveals that *S. frugiperda* herbivory differentially regulates two cytochrome P450 enzymes involved in this reaction, uncovering a temporal optimization strategy in which different enzymes dominate at successive stages of the defense response. This metabolic shift is consistent with the distinct physicochemical properties of the two compounds, as 10-HCPT exhibits relatively higher water solubility than the nearly insoluble CPT, thereby facilitating cell-to-cell transport and accumulation in epidermal structures such as trichomes. Such spatial separation of synthesis and storage reflects a common principle in plant secondary metabolism that minimizes autotoxicity while positioning defenses at the plant–insect interface. Specifically in *C. acuminata*, the expression of these cytochrome P450 genes is induced exclusively by herbivory rather than wounding alone, indicating that salivary effectors instead of physical injury drive the metabolic shift.

**Figure 4:**
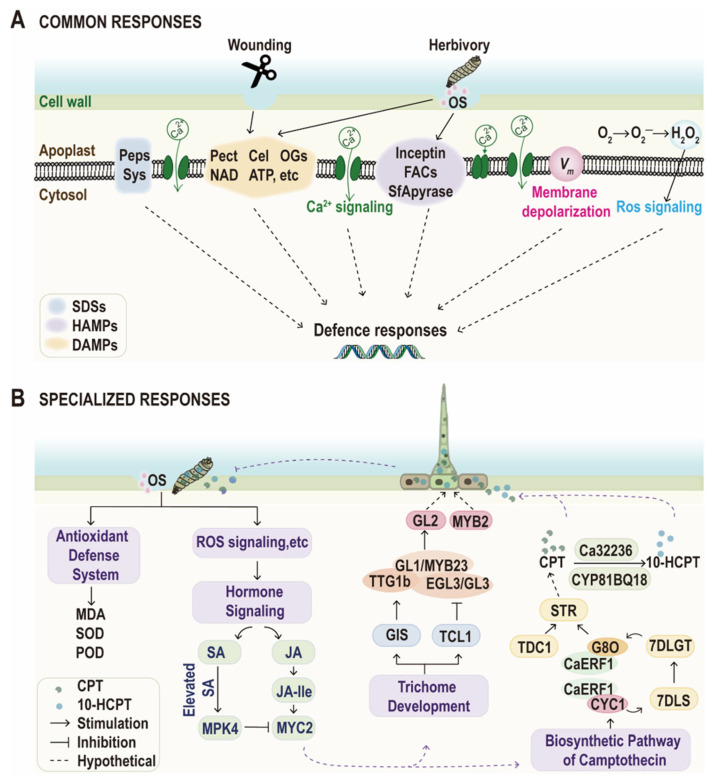
Proposed model of stress responses in *C. acuminata*. (**A**) Common defense activation pathway triggered by mechanical wounding and insect herbivory. (**B**) Specialized defense mechanism specifically induced by insect herbivory in *C. acuminata*. Solid arrows denote experimentally confirmed causal relationships or direct molecular interactions. Dashed arrows indicate putative or hypothesized promoting effects that require further validation. Horizontal arrows represent inhibitory or suppressive regulatory interactions. Abbreviations: Peps, peptides 1–6; Sys, systemin; Cel, cellulose; OG, oligogalacturonide; NAD, nicotinamide adenine dinucleotide; ATP, adenosine 5′-triphosphate; FAC, fatty acid–amino acid conjugate; HAMP, herbivore-associated molecular pattern; DAMP, damage-associated molecular pattern; SDS, secondary danger signal.

As a potent DNA topoisomerase I inhibitor, CPT exhibits significant cytotoxicity that provides plants with a robust defense against diverse biotic threats. Despite its structural complexity and high biosynthetic cost, the convergent evolution of CPT across 43 plant species in eight distantly related families, including Nyssaceae and Rubiaceae, underscores its indispensable role in broad spectrum defense. In response to herbivory, plants typically initiate a suite of conserved physiological adjustments, such as modulating the activities of SOD, CAT, and POD, while altering MDA levels to mitigate oxidative stress (Figure 4A). Beyond these generalized responses, certain species have evolved specialized defense strategies. *C. acuminata*, a prominent producer of CPT, exemplifies this specialization (Figure 4B). While activating common stress responses, it simultaneously coordinates multiple defensive dimensions, including CPT biosynthesis, trichome development, and the metabolic conversion of CPT to 10-HCPT, to enhance resistance.

By characterizing the multidimensional defense system comprising both physical and chemical barriers in *C. acuminata* during herbivory by *S. frugiperda*, this study demonstrates how plants integrate conserved and specialized mechanisms to achieve highly efficient protection against biotic stress. These findings provide a comprehensive framework for understanding the evolutionary and physiological strategies that enable plants to optimize their survival in hostile environments.

## 4. Materials and Methods

### 4.1. Plant Materials and Growth Conditions

Uniform six-month-old *C. acuminata* seedlings approximately 30 cm in height and bearing eights leaves were cultivated in nutrient soil (Shanghai Qinli Network Technology Co., Ltd., Shanghai, China) within a growth chamber. Prior to the experiments, the growth conditions were maintained at 25 °C with a relative humidity of 60% under a 16-h photoperiod.

### 4.2. Insect Rearing, Plant Treatments, and Sample Collection

A laboratory population of *S. frugiperda* was established from individuals collected in cornfields in Conghua District, Guangzhou, Guangdong, China, and maintained on a laboratory-prepared artificial diet for over 15 generations in an incubator at 25 ± 1 °C, 70 ± 5% relative humidity, and a 12 h photoperiod. For herbivory treatment, two fourth-instar larvae were confined to the two upper fully expanded leaves of each seedling which typically possessed eight true leaves at this developmental stage using a mesh cage. Mechanical wounding was applied to the corresponding leaves of separate plants using a sterile punch to simulate physical damage. To ensure consistent environmental conditions, both the mechanically wounded and untreated control plants were enclosed in identical mesh cages.

To conduct subsequent physiological, biochemical, and gene expression analyses, leaves were collected when the damaged area reached approximately 25% (12 h) and 50% (24 h) of the total leaf area. For each treatment at each damage stage, 30 independent plants were divided into three groups of 10. The leaves within each group were then pooled to constitute a single biological replicate, yielding three independent replicates per condition. All samples were immediately flash-frozen in liquid nitrogen and stored at −80 °C.

Photographs of treated leaves were taken prior to sampling, and total leaf area as well as damaged area were measured by tracing leaf margins using ImageJ 1.44p software. The percentage of leaf damage was calculated as (damaged area/total leaf area) × 100%. For trichome observation, newly emerged leaves from treated plants were collected, mounted on slides, and imaged using an IX73 inverted microscope (Olympus Corporation, Tokyo, Japan).

To evaluate larval survival in vivo *C. acuminata*, fourth-instar *S. frugiperda* larvae were continuously confined on intact seedling leaves under the same environmental conditions described above. Larval survival was monitored over a 24 h period, and mortality was recorded when larvae showed no movement upon gentle stimulation with a soft brush.

### 4.3. RNA Isolation and Real-Time qPCR

To assess the expression levels of CPT biosynthetic and trichome development-related genes in *C. acuminata*, total RNA was extracted from each treatment using TRIzol reagent (Sangon Biotech (Shanghai, China) Co., Ltd.) according to the manufacturer’s instructions. The isolated RNA was reverse-transcribed into cDNA using a commercial reverse transcription kit (Takara Bio, Dalian, China). Subsequent real-time qPCR was conducted on a LightCycler^®^ 480 II system (Roche Diagnostics, Basel, Switzerland) in a 20-µL reaction volume. Each reaction mixture contained SYBR Green Supermix (TransGen Biotech, Beijing, China), 0.2 µM of each specific primer (Table S1), and a cDNA template equivalent to 5 ng of total RNA. The thermal cycling parameters included an initial denaturation at 95 °C for 1 min, followed by 40 cycles of 95 °C for 20 s, 50 °C for 15 s, and 72 °C for 15 s. Relative gene expression was calculated from the cycle threshold (Ct) values using the 2^−ΔΔ*Ct*^ method.

### 4.4. Determination of Chlorophyll Contents, Antioxidant Enzyme Activities, and Malondialdehyde (MDA) Contents

*C. acuminata* leaves were harvested and gently rinsed with deionized water to remove dust. For determining antioxidant enzyme activities in the collected samples, 1 g of fresh leaves were ground in 10 mL of 50 mM potassium phosphate buffer (1 mM EDTA, 5% polyvinylpolypyrrolidone, 2 mM phenylmethylsulphonyl fluoride, and 0.5% triton X-100), and then kept at 4 °C for 10 min. The supernatants were obtained by centrifugation and used for measuring the activities of superoxide dismutase (SOD), catalase (CAT), and peroxidase (POD) by standard procedures [53,54].

MDA contents in *C. acuminata* leaves were detected according to the method developed previously [55]. Then, 0.5 g of fresh leaves of each treatment was crushed with 100 mM phosphate buffer, and the extract was collected by centrifugation. Trichloroacetic acid (TCA) and thiobarbituric acid (TBA) were then added with a final concentration of 5% to the extract. After incubation at 100 °C for 10 min, the supernatants were obtained by centrifugation and used to measure their absorbance values at 532 nm by an EnSight™ multimode plate reader (PerkinElmer, Waltham, MA, USA) to determine MDA contents.

Soluble sugar content was quantified using the anthrone colorimetric method [56]. Contents of chlorophyll in *C. acuminata* leaves were measured using the method developed previously [57].

### 4.5. Sample Preparation and HPLC Quantification of CPT and 10-HCPT in Leaves and Trichomes

Samples were prepared according to a previously reported method [26]. Briefly, *C. acuminata* leaves were dried at 45 °C for 48 h. Then, 0.2 g of each sample was extracted with 1.0 mL of methanol for 10 min. The extract was centrifuged at 12,000 rpm for 10 min, and the supernatant was filtered through a 0.22 μm filter. For trichome isolation, leaves were briefly immersed in liquid nitrogen for approximately 30 s while being held with forceps. Trichomes on the leaf surface were then rapidly detached using a brush and collected into a precooled beaker for subsequent analysis. The isolated trichomes were then extracted with 5 mL of methanol, centrifuged, and filtered similarly for HPLC analysis. The contents of CPT and 10-HCPT were determined using an Agilent 1260 series HPLC system (Agilent Technologies, Santa Clara, CA, USA) equipped with a C18 column (250 mm × 4.6 mm, 5 μm). The column temperature was maintained at 30 °C, and the injection volume was 10 μL. Separation was achieved by gradient elution with a mobile phase consisting of (A) acetonitrile and (B) water at a flow rate of 1.0 mL/min. The gradient program was as follows: 0–12 min, 20–26% A; 12–20 min, 26–40% A; 20–25 min, 40–20% A; 25–30 min, 20% A (isocratic). Commercially available CPT and 10-HCPT (Sigma-Aldrich, St. Louis, MO, USA) were used as reference standards to establish calibration curves (Figure S2).

### 4.6. Statistical Analysis

Data are presented as mean ± standard deviation (SD). Statistical analyses were performed using SPSS 20.0 (IBM, Armonk, NY, USA). Prior to analysis, normality and homogeneity of variances were assessed using the Shapiro–Wilk and Levene’s tests, respectively. When necessary, data were transformed or analyzed using appropriate non-parametric methods. For study assessing the interaction between damage types and severities, differences among treatments were analyzed using two-way analysis of variance (ANOVA). Damage type (mechanical wounding and herbivory) and damage level (0%, 25%, and 50% leaf area loss) were treated as fixed factors, and the full model included the interaction term. Following significant ANOVA results, post hoc multiple comparisons were performed using Tukey–Kramer tests (*p* < 0.05), with statistical significance denoted by different letters. For independent two-group comparisons, pairwise differences were analyzed separately using Student’s *t*-tests, with significance indicated by asterisks (* *p* < 0.05, ** *p* < 0.01, *** *p* < 0.001). All statistical analyses were based on three independent biological replicates (*n* = 3), each consisting of pooled tissue samples collected from 10 independently treated plants.

## 5. Conclusions

Plants have evolved complex multidimensional defense systems to withstand external stresses, thereby ensuring their survival and sustained growth under adverse conditions. CPT is a specialized alkaloid with documented defensive and pharmaceutical activities and has been identified in 43 species across eight plant families. The present study examined the defense responses of the CPT-producing plant *C. acuminata* to herbivory by *S. frugiperda* larvae. The results demonstrated that larval herbivory modified the activities of SOD, CAT, and POD while elevating MDA content as part of the oxidative stress response. In addition, the infestation triggered CPT biosynthesis alongside trichome development, thereby establishing both chemical and physical barriers to biotic stress. Larval feeding stress further promoted the bioconversion of CPT to 10-HCPT, with the resultant increase in water solubility from hydroxylation potentially facilitating the systemic redistribution of these defensive compounds within the plant.

## Data Availability

The original contributions presented in this study are included in the article. Further inquiries can be directed to the corresponding author.

## Supplementary Materials

The following supporting information can be downloaded at: https://www.mdpi.com/article/10.3390/plants15121796/s1, Table S1. Primers used in this study. Figure S1. Trichome density correlates with CPT content in *C. acuminata* young leaves. (A) Trichome density at different leaf developmental stages. (B) Linear relationship between trichome index and CPT content of young leaves of *C. acuminata*. (C) Linear relationship between trichome index and 10-HCPT content of young leaves of *C. acuminata*. Figure S2. The HPLC chromatograms of CPT and 10-HCPT.
