# Supplementary material for: Activation of Multidimensional Defenses in Camptotheca acuminata Seedlings Against Spodoptera frugiperda Larvae
Source: Plants (Basel). 2026 Jun 11;15(12):1796. doi: 10.3390/plants15121796 (PMC13307388; doi:10.3390/plants15121796)
Supplement: Supplementary file 1 [file plants-15-01796-s001.zip › plants-4255371-supplementary.pdf]

Supporting Information

Activation of Multidimensional Defenses in *Camptotheca acuminata* seedlings against *Spodoptera frugiperda* Larvae

Wenhui Ma[1,†], Chunhao Chang[2,†], Jianing Cheng[3], Yanyan Wang[1], Xiaoxiao Gao[1,*] and Fang Yu[1,3,*]

[1] School of Biological Engineering, Dalian Polytechnic University, Dalian 116034, China

[2] College of Horticulture and Forestry, Tarim University, Alar, Xinjiang 843300, China

[3] College of Bioscience and Biotechnology, Shenyang Agricultural University, Shenyang 110866, China

* Correspondence: xxgao@dlpu.edu.cn (X.G.); fyu@syau.edu.cn or fyu0506@gmail.com (F.Y.)

† These authors contributed equally to this work.

Table S1. Primers used in this study

Gene name	Forward primer sequence (5'to3')	Reverse primer sequence (5'to3')
CaACT3	AGTCGATTGCCAGGACAGTC	CCAAATCCACAATGACGAAG
CaMYC2	TTCCCATAGTCAGGAAATCTCGAAA	CTGGTAAACAAGCTCTGTTTTTCGT
CaNPR1	CGTTTCTTCCCTCGTTGTT	ATGGATGTCGAAGACGATGA
CaTTG1b	AAGGCCAACCCGAATCTATC	TCTCCAGAGGATGCGAGCAG
CaMYB23a	CTCCTTGGAAACAGGTGGTC	CCCGGACACTTTCACTACTC
CaGIS1	CCACCTTTCTGTCCTCCTTT	AGGTGCAGGGCGAATAAAGG
CaMYB2	ATGTGTTCAATGGACAACGC	TAATTAAGCACGGAAATCGA
CaGL2	AATCACTCAGACCCCACCCT	CTCGGAAAATCCCAGCGAGA
CaTCL1	TTTTATCTTCTGCTCCTCCTCT	AACTCCCACTCAATACTGCTCA
CaTDC1	AACATACGCTCCATCCCG	CGTCCAAATAGTGCCGAAAT
CaCYC1	TTCTGTGGTGGGTCGTGTAT	ATTGTTGTCCAGTGCAAAGG
Ca7DLS	CCTATTTGGATCTGGGAGAC	TCTCTGGGCTGACATGACTAT
Ca7DLGT	CATCATATTGTAATTTGGACCG	AACCAAATAGCTGCAGGAAT
CaG8O	TCAACCTCTTCGTGCTGTTC	ATAGAACGAGAACTCCGACG
CaSTR2	ACTTGAGCCAACTCAGTCAGT	GGGACCTGTCAGCCAATA
CaERF1	CAATCGAAATCAATCGTTGC	GGCGTGTCGTAAGATCCTAA
Ca32236	CTCTGCCTTTCATTGTTCACT	CAAACGGACTTGGTGGTAAC
CaCYP81BQ18	TTGCGTAGATTGGCTGTTGT	AGCCATAGAAGCACCTGGAC

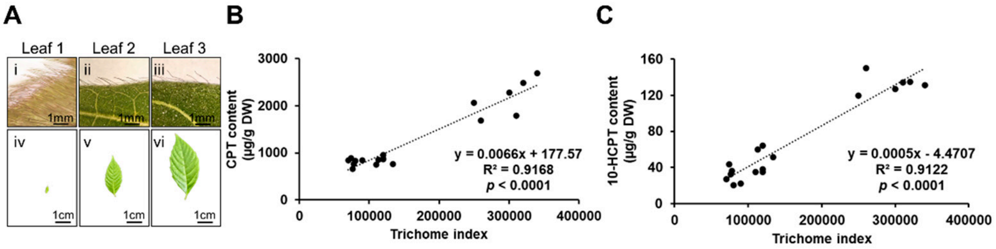

Figure S1. Trichome density correlates with camptothecin alkaloid content in *C. acuminata* young leaves. **(A)** Trichome density at different leaf developmental stages. **(B)** Linear relationship between trichome index and CPT content of young leaves of *C. acuminata***.** **(C)** Linear relationship between trichome index and 10-HCPT content of young leaves of *C. acuminata***.** *p*-value of less than 0.05 was considered statistically significant, (n=18).

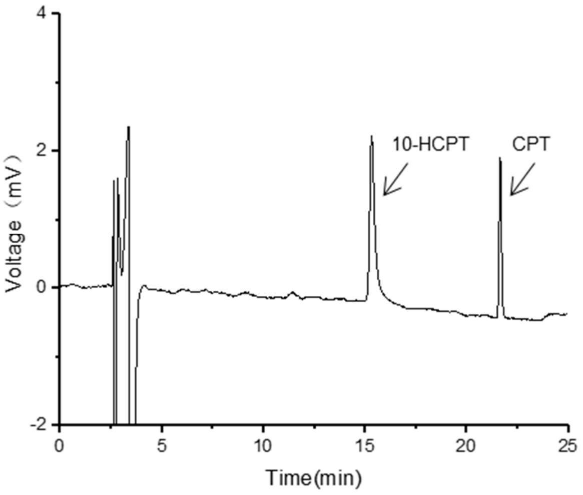

Figure S2. The HPLC chromatograms of CPT and 10-HCPT.
